# Clinical Pregnancy Outcomes Following Blastocyst Transfer Using Media with Different Hyaluronan Concentrations: A Retrospective Cohort Study

**DOI:** 10.3390/life16101750

**Published:** 2026-10-19

**Authors:** Mustafa Kemal Özel, Fırat Şahin, Fırat Aşir, Mehmet Turan Çetin

**Affiliations:** 1 Faculty of Health Sciences, Osmaniye Korkut Ata University, Osmaniye 80000, Türkiye; 2 Division of Infertility, Turan Çetin IVF Center, Adana 01360, Türkiye; 3 Department of Histology and Embryology, Faculty of Medicine, Dicle University, Diyarbakır 21280, Türkiye

**Keywords:** hyaluronan, embryo transfer medium, blastocyst transfer, clinical pregnancy, hyaluronan concentration, assisted reproductive technology

## Abstract

**Background and Objectives:** Hyaluronan-enriched embryo transfer media have been proposed to improve implantation and reproductive outcomes; however, their clinical benefit remains uncertain, particularly in frozen embryo transfer cycles. This study aimed to compare clinical pregnancy outcomes following blastocyst transfer using two commercially available media with different hyaluronan concentrations. **Materials and Methods:** This retrospective cohort study included 421 blastocyst transfer cycles performed at a single IVF center between November 2024 and April 2026. Seventy-two cycles used a hyaluronan-enriched transfer medium (EmbryoGlue), and 349 used a medium with a lower hyaluronan concentration (SAGE 1-Step). The primary outcome was clinical pregnancy, defined as an intrauterine pregnancy with fetal cardiac activity. Exploratory multivariable logistic regression was performed adjusting for maternal age and transfer type. **Results:** Clinical pregnancy occurred in 21/72 cycles (29.2%) in the hyaluronan-enriched medium group and 127/349 cycles (36.4%) in the lower-hyaluronan medium group (*p* = 0.30). The use of the hyaluronan-enriched medium was not significantly associated with clinical pregnancy after adjustment for maternal age and transfer type (aOR = 0.77, 95% CI: 0.44–1.34, *p* = 0.352). Increasing maternal age was associated with lower odds of clinical pregnancy (aOR = 0.95 per year, 95% CI: 0.91–0.98, *p* = 0.002), whereas transfer type was not (aOR = 1.14, 95% CI: 0.59–2.21, *p* = 0.688). No significant differences were observed in either maternal age subgroup. **Conclusions:** In this predominantly frozen blastocyst-transfer cohort, no statistically significant difference in clinical pregnancy rates was observed between the hyaluronan-enriched and lower-hyaluronan transfer medium groups. Given the retrospective, non-randomized design, differences in medium composition beyond hyaluronan concentration, and potential residual confounding, these findings should not be interpreted as evidence of equivalence or as demonstrating the absence of a clinically relevant effect. Prospective randomized studies evaluating live birth are warranted.

## 1. Background

Infertility is a major global health issue, with approximately one in six adults experiencing infertility during their lifetime [1]. Beyond its reproductive consequences, infertility may impose substantial psychological and social burdens on affected individuals and couples [2]. Assisted reproductive technology has substantially advanced the management of infertility, with developments such as modern cryopreservation techniques contributing to major changes in clinical practice [3]. Nevertheless, successful implantation remains a critical determinant of treatment success, and favorable embryo morphology does not invariably ensure implantation [4,5]. This variability reflects the complex interaction among embryo developmental competence, endometrial receptivity, and factors related to the embryo transfer procedure itself [5,6,7].

Embryo transfer is a critical step in in vitro fertilization (IVF) treatment, as procedural factors may influence the likelihood of successful implantation even when a viable embryo is available [6,7]. In addition to embryo quality and endometrial receptivity, several technical aspects of embryo transfer, including catheter characteristics, ultrasound guidance, transfer difficulty, uterine contractility, the presence of blood or mucus on the catheter, and the composition of the transfer medium, have been investigated as potential determinants of treatment outcome [6,7]. Although efforts have been made to standardize embryo transfer procedures, optimization of the transfer environment remains an area of clinical interest, particularly through interventions intended to facilitate embryo–endometrial interaction without substantially increasing procedural complexity [6,7].

Hyaluronan is a naturally occurring high-molecular-weight glycosaminoglycan and an important component of the extracellular matrix within the female reproductive tract [8]. It is present in reproductive tissues and fluids, including the endometrium and follicular fluid [8,9,10]. Hyaluronan is involved in extracellular matrix organization and tissue remodeling, including dynamic changes within the endometrium during the menstrual cycle [10,11]. During the peri-implantation period, hyaluronan has been proposed to contribute to endometrial receptivity and embryo–endometrial interactions through extracellular matrix organization and signaling pathways involving hyaluronan receptors such as CD44 [11]. These biological properties have provided the rationale for incorporating hyaluronan into embryo transfer media as a potential strategy to support implantation [11,12].

Hyaluronan-enriched embryo transfer media have been introduced into clinical practice with the aim of improving implantation and reproductive outcomes [12,13]. Their effects on implantation, clinical pregnancy, ongoing pregnancy, and live birth have been evaluated in randomized controlled trials and systematic reviews, with variable findings across studies and clinical settings [12,13]. Although pooled evidence has suggested a potential improvement in reproductive outcomes with hyaluronan-enriched transfer media, this benefit has not been consistently demonstrated across transfer settings, particularly in frozen embryo transfer cycles [12,13]. Consequently, the clinical value of hyaluronan-enriched transfer medium use may differ according to the clinical and embryo transfer setting [12,13].

Despite the substantial body of evidence on hyaluronan-enriched transfer media, uncertainty remains regarding their incremental clinical value in contemporary embryo transfer practice, particularly in frozen embryo transfer settings [12,13]. The available evidence encompasses heterogeneous patient populations, embryo developmental stages, and transfer settings, which may affect the applicability of pooled findings to specific clinical scenarios [12,13]. Evaluating hyaluronan-enriched transfer media specifically in blastocyst-transfer cohorts predominantly comprising frozen embryo transfers may therefore provide complementary real-world evidence regarding their routine clinical use.

Against this background, the present study aimed to compare clinical pregnancy outcomes following blastocyst transfer using two commercially available transfer media with different hyaluronan concentrations: EmbryoGlue^®^, a hyaluronan-enriched transfer medium, and SAGE 1-Step^TM^, a medium containing a lower concentration of hyaluronan. We hypothesized that clinical pregnancy rates would differ between the two transfer medium groups. In addition, an exploratory multivariable analysis was performed to assess this association after adjustment for maternal age and transfer type (fresh versus frozen embryo transfer).

## 2. Materials and Methods

This retrospective observational cohort study included women undergoing single blastocyst transfer at the Prof. Dr. Turan Çetin IVF Center between November 2024 and April 2026. Eligible patients were women undergoing fresh or frozen blastocyst transfer who had undergone pre-transfer uterine cavity evaluation by hysterosalpingography or hysteroscopy and had complete clinical and embryo transfer data. Only one embryo transfer cycle per woman was included in the analysis.

Exclusion criteria included severe oligozoospermia or the use of surgically retrieved sperm, maternal body mass index (BMI) > 30 kg/m^2^, chronic medical diseases, hyperprolactinemia, thyroid disorders, endometriosis, hydrosalpinx, recurrent pregnancy loss, recurrent implantation failure, hypogonadotropic hypogonadism, day 2–4 embryo transfers, absence of pre-transfer uterine cavity evaluation by hysterosalpingography or hysteroscopy, and incomplete clinical or embryo transfer records.

**Figure 1:**
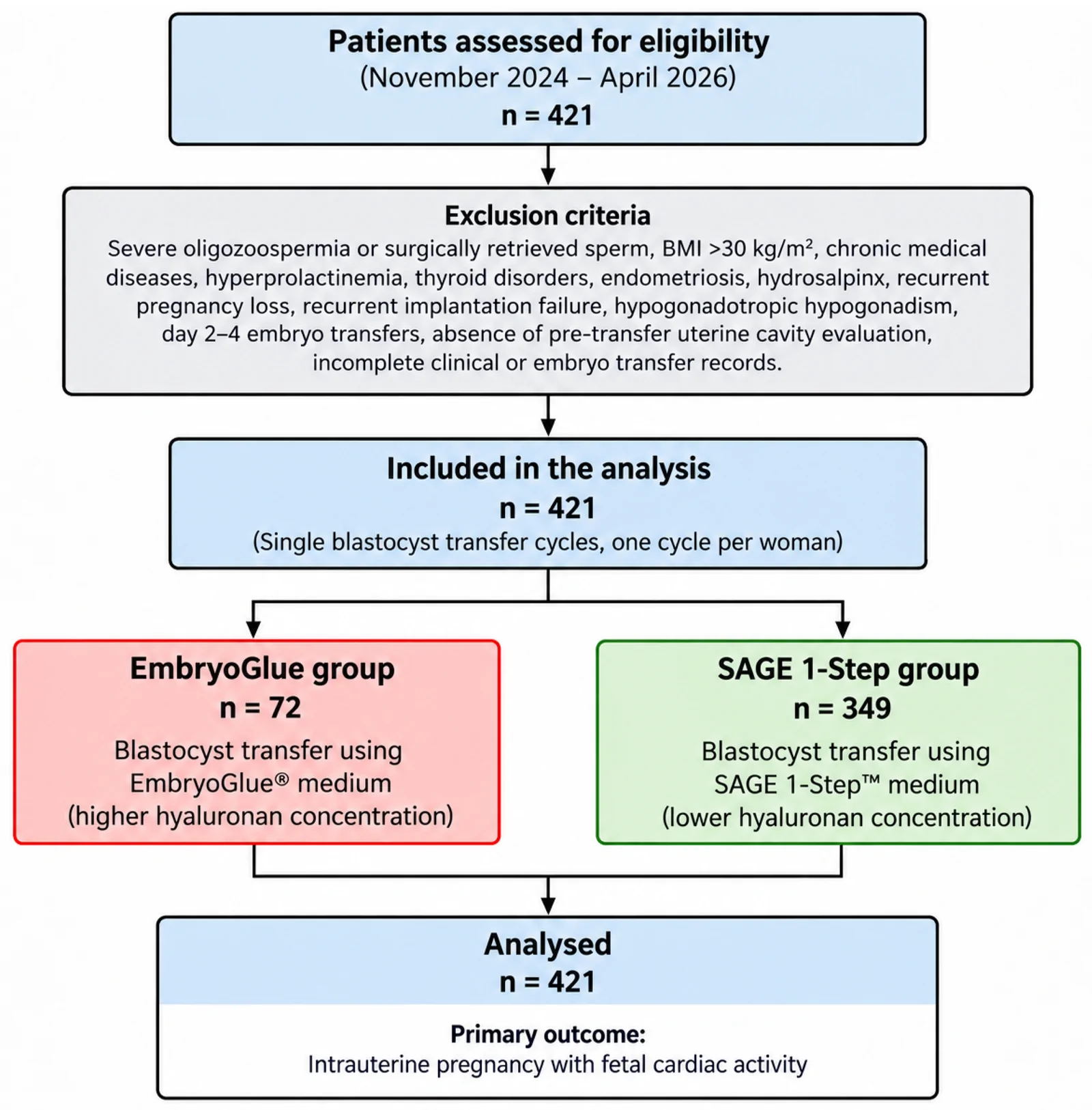
Flowchart of the study population and distribution by embryo transfer medium. A total of 421 women undergoing single blastocyst transfer were included: 72 in the EmbryoGlue® group and 349 in the SAGE 1-Step^TM^ group.

During the study period, 421 blastocyst transfer cycles from 421 women met the eligibility criteria and were included in the final analysis. Clinical pregnancy outcome data were available for all included cycles. The study population comprised 72 cycles in the hyaluronan-enriched transfer medium group (EmbryoGlue) and 349 cycles in the lower-hyaluronan transfer medium group (SAGE 1-Step). The study population and distribution of patients between the two transfer-medium groups are presented in Figure 1.

The study protocol was approved by the Scientific Research Ethics Committee of Çukurova University Faculty of Medicine on 5 June 2026 (Meeting No. 167, Decision No. 65) and was conducted in accordance with the principles of the Declaration of Helsinki.

### 2.1. Ovarian Stimulation and IVF Laboratory Procedures

Oocytes were obtained following controlled ovarian stimulation using a gonadotropin-releasing hormone (GnRH) antagonist protocol. Cycles involving progestin-primed ovarian stimulation were excluded. Final oocyte maturation was triggered using either human chorionic gonadotropin (hCG) or triptorelin (Gonapeptyl^®^). Transvaginal ultrasound-guided oocyte retrieval was performed 34–36 h after trigger administration.

Semen samples were collected by masturbation following three days of sexual abstinence. Sperm preparation was performed using density-gradient centrifugation at 800 rpm for 8 min. All oocytes were inseminated using intracytoplasmic sperm injection (ICSI), and cycles involving conventional IVF were excluded.

Embryos were cultured using a single-step culture medium (SAGE 1-Step^TM^; CooperSurgical, Trumbull, CT, USA) in MIRI^®^ incubators (Esco Medical, Singapore) at 37 °C under reduced oxygen conditions (5% O_2_). The nominal CO_2_ concentration was 6.1%, with minor adjustments based on routine culture-medium pH measurements to maintain a target pH of approximately 7.28. Only blastocyst-stage embryo transfers were included in the analysis. Blastocyst morphology was evaluated using the Gardner grading system, which assesses blastocyst expansion stage, inner cell mass, and trophectoderm morphology [14].

For descriptive analysis, blastocysts were categorized according to expansion stages 3, 4, and 5 and morphological grades AA, AB, and BB, as recorded in the embryology laboratory database. These characteristics were summarized by transfer medium group but were not included in the multivariable logistic regression model.

Embryos designated for cryopreservation underwent vitrification using ORIGIO vitrification kits. Frozen blastocysts were subsequently warmed using ORIGIO warming kits according to the center’s routine laboratory protocols.

### 2.2. Transfer Medium and Embryo Transfer Protocol

In frozen embryo transfer cycles, warmed blastocysts were initially maintained in SAGE 1-Step^TM^ (CooperSurgical, USA). In fresh transfer cycles, blastocysts were maintained in SAGE 1-Step^TM^ before the transfer-medium step. In the lower-hyaluronan medium group, blastocysts remained in SAGE 1-Step^TM^ until embryo transfer. In the hyaluronan-enriched medium group, blastocysts were transferred from SAGE 1-Step^TM^ to EmbryoGlue^®^ (Vitrolife, Gothenburg, Sweden), a hyaluronan-enriched embryo transfer medium, approximately 1 h before embryo transfer, where they remained until the transfer procedure. Both media contain hyaluronan, with a concentration of 0.5 mg/mL in EmbryoGlue^®^ and 0.1 mg/mL in SAGE 1-Step^TM^. As the two media also differ in other formulation components, the study compared two commercially available transfer media rather than isolating the effect of hyaluronan concentration. All patients underwent single embryo transfer, with one blastocyst transferred per cycle.

The decision to use EmbryoGlue^®^ was made by the treating physician on the day of embryo transfer and was not based on patient prognosis or previous treatment outcomes. On days with multiple embryo transfers, the hyaluronan-enriched medium was generally used for the first scheduled transfer rather than being selected according to specific patient or embryo characteristics. The use of the hyaluronan-enriched transfer medium was based on its proposed implantation-supporting properties and the available evidence and ESHRE recommendations regarding hyaluronan supplementation of embryo transfer media [13]. Embryo transfers were performed under ultrasound guidance using the same type of embryo transfer catheter in both groups. All patients received standardized luteal phase support consisting of vaginal progesterone (600–800 mg/day), oral estradiol (6 mg/day), and acetylsalicylic acid (100 mg/day), regardless of whether fresh or frozen embryo transfer was performed. Apart from the transfer medium protocol, the embryo transfer procedure and peri-transfer management were comparable between groups.

### 2.3. Clinical Pregnancy Assessment

Serum β-hCG was measured after embryo transfer according to the center’s routine follow-up protocol. Patients with serum β-hCG levels exceeding 2000 IU/L were invited for transvaginal ultrasonography to confirm the presence of an intrauterine gestational sac. A follow-up transvaginal ultrasound examination was performed approximately 2 weeks later to assess crown–rump length and fetal cardiac activity. Clinical pregnancy was defined as the presence of an intrauterine pregnancy with fetal cardiac activity confirmed by transvaginal ultrasonography.

### 2.4. Statistical Analysis

Statistical analyses were performed using IBM SPSS Statistics version 25 (IBM Corp., Armonk, NY, USA). The distribution of continuous variables was assessed using the Shapiro–Wilk test. Continuous variables were expressed as mean ± standard deviation (SD), and categorical variables as frequencies and percentages. Comparisons between the hyaluronan-enriched and lower-hyaluronan medium groups were performed using the independent-samples *t*-test for continuous variables and the chi-square test or Fisher’s exact test for categorical variables, as appropriate.

As this was a retrospective cohort study, no a priori sample size calculation was performed. The sample size was determined by the number of eligible patients with available records during the study period. An exploratory sensitivity analysis was conducted using a normal approximation for the comparison of two independent proportions, with group sizes of 72 and 349, a two-sided significance level of 0.05, 80% statistical power, and a reference clinical pregnancy rate of 36.4% in the lower-hyaluronan medium group. This analysis was intended to characterize the magnitude of between-group differences detectable with the available sample size rather than to determine sample size retrospectively.

The primary outcome was clinical pregnancy. An exploratory multivariable logistic regression analysis was performed to evaluate the association between transfer medium type (EmbryoGlue versus SAGE 1-Step) and clinical pregnancy after adjustment for maternal age and transfer type (fresh versus frozen embryo transfer). Adjusted odds ratios (aORs) with 95% confidence intervals (CIs) were reported. Although blastocyst morphological characteristics were available for descriptive analysis, these variables were not included in the multivariable logistic regression model. Other potentially relevant clinical covariates were unavailable. Therefore, the adjusted model was considered exploratory and was not intended to establish an independent treatment effect or causal relationship.

Subgroup analyses were additionally performed according to maternal age (≤35 and >35 years). All statistical tests were two-sided, and a *p*-value < 0.05 was considered statistically significant. The final analytical dataset contained complete information on maternal age, embryo transfer type, and clinical pregnancy outcome for all 421 women. Accordingly, no missing-data imputation was required, and all 421 observations were included in the multivariable logistic regression analysis.

## 3. Results

All 421 cycles involved single blastocyst transfer, including 72 cycles in the hyaluronan-enriched medium group and 349 cycles in the lower-hyaluronan medium group. Mean maternal age was 33.29 ± 6.76 years in the hyaluronan-enriched medium group and 31.85 ± 5.96 years in the lower-hyaluronan medium group (*p* = 0.068). Frozen embryo transfer cycles accounted for 90.3% (65/72) and 87.1% (304/349) of cycles in the hyaluronan-enriched and lower-hyaluronan medium groups, respectively (*p* = 0.48) (Table 1). Clinical pregnancy occurred in 21 of 72 cycles (29.2%) in the hyaluronan-enriched medium group and 127 of 349 cycles (36.4%) in the lower-hyaluronan medium group; the difference was not statistically significant (*p* = 0.30).

Blastocyst expansion stages and morphological grades according to the Gardner classification are presented in Table 2. In the hyaluronan-enriched medium group, stage 3, stage 4, and stage 5 blastocysts accounted for 54.2%, 30.6%, and 15.3% of transfers, respectively, compared with 51.6%, 26.6%, and 21.8% in the lower-hyaluronan medium group. Regarding blastocyst morphology, AA, AB, and BB grades accounted for 37.5%, 36.1%, and 26.4% of transfers in the hyaluronan-enriched medium group, respectively, compared with 34.1%, 39.8%, and 26.1% in the lower-hyaluronan medium group.

In the exploratory multivariable logistic regression analysis adjusted for maternal age and transfer type, use of the hyaluronan-enriched medium, compared with the lower-hyaluronan medium, was not significantly associated with clinical pregnancy (aOR = 0.77, 95% CI: 0.44–1.34, *p* = 0.352). Maternal age was inversely associated with clinical pregnancy, with a 5% decrease in the odds of clinical pregnancy per additional year of age (aOR = 0.95 per year, 95% CI: 0.91–0.98, *p* = 0.002). Transfer type was not significantly associated with clinical pregnancy (FET vs. fresh transfer: aOR = 1.14, 95% CI: 0.59–2.21, *p* = 0.688) (Table 3).

In the subgroup analysis according to maternal age, clinical pregnancy rates did not differ significantly between the hyaluronan-enriched and lower-hyaluronan medium groups in either age category. Among women aged ≤35 years, clinical pregnancy occurred in 14 of 44 cycles (31.8%) in the hyaluronan-enriched medium group and 100 of 251 cycles (39.8%) in the lower-hyaluronan medium group (OR = 0.71, 95% CI: 0.36–1.40, *p* = 0.313). Among women aged >35 years, clinical pregnancy occurred in 7 of 28 cycles (25.0%) in the hyaluronan-enriched medium group and 27 of 98 cycles (27.6%) in the lower-hyaluronan medium group (OR = 0.88, 95% CI: 0.33–2.30, *p* = 0.789) (Table 4).

The exploratory sensitivity analysis indicated that the available sample size provided approximately 80% power to detect an increase in clinical pregnancy rate from 36.4% to 54.4% (an absolute difference of 18.0 percentage points) or a decrease from 36.4% to 20.2% (an absolute difference of 16.2 percentage points), using a two-sided significance level of 0.05. Therefore, the study had limited statistical power to detect smaller between-group differences.

## 4. Discussion

In this retrospective cohort of women undergoing single blastocyst transfer, no statistically significant difference in clinical pregnancy rates was observed between the hyaluronan-enriched and lower-hyaluronan transfer medium groups. Clinical pregnancy rates were 29.2% in the hyaluronan-enriched medium group and 36.4% in the lower-hyaluronan medium group, and no statistically significant differences were observed in either maternal age subgroup. In the exploratory multivariable analysis adjusted for maternal age and transfer type, the use of the hyaluronan-enriched medium compared with the lower-hyaluronan medium was not significantly associated with clinical pregnancy, whereas increasing maternal age was associated with lower odds of clinical pregnancy.

This finding is consistent with the well-established decline in reproductive potential with advancing maternal age, primarily attributable to reduced oocyte competence and increased embryonic aneuploidy rates [15,16]. Although maternal age was included in the multivariable model, adjustment for age alone may not adequately account for differences in embryo competence between the study groups. Blastocyst morphological characteristics were available for descriptive analysis but were not included in the adjusted model, and preimplantation genetic testing data were unavailable.

Several factors may explain the absence of a statistically significant difference in clinical pregnancy rates between the hyaluronan-enriched and lower-hyaluronan transfer medium groups in the present cohort. Implantation is a multifactorial process influenced by embryo competence, endometrial receptivity, and transfer-related factors, and the potential contribution of the transfer medium may therefore be relatively modest [5,7]. Furthermore, most cycles in our cohort were frozen embryo transfers, and the clinical effect of hyaluronan-enriched media may vary according to transfer setting and patient characteristics [12,13]. Importantly, both transfer media contain hyaluronan and differ in other formulation components. Consequently, the present findings cannot be attributed specifically to differences in hyaluronan concentration or interpreted as evidence of the isolated effect of hyaluronan. Although the choice of transfer medium was not based on patient prognosis or previous treatment outcomes, its non-randomized allocation introduces the possibility of selection bias and unmeasured confounding.

Previous randomized studies and systematic reviews have reported inconsistent findings, with some demonstrating improvements in implantation or pregnancy outcomes and others showing no clear benefit [12,13,17]. A systematic review and meta-analysis by Heymann et al. suggested that hyaluronan-enriched transfer media may improve reproductive outcomes in certain autologous treatment settings; however, considerable clinical and methodological heterogeneity was observed across the included studies [18]. Furthermore, the available evidence suggests that the effects of hyaluronan-enriched transfer media may vary according to embryo developmental stage, transfer type (fresh versus frozen), and patient characteristics [13,17,18].

More recent evidence further illustrates the uncertainty regarding the clinical value of hyaluronan-enriched transfer media in frozen embryo transfer cycles. In a randomized double-blind controlled trial, Yung et al. found no improvement in live birth or clinical pregnancy rates with hyaluronan-enriched transfer medium in frozen embryo transfer cycles [19]. In contrast, Yan et al. reported a higher clinical pregnancy rate among women with recurrent implantation failure undergoing frozen embryo transfer, although a significant improvement in live birth was not demonstrated [20]. Earlier studies also yielded heterogeneous findings. Korošec et al. found similar overall pregnancy rates following single fresh and frozen-thawed blastocyst transfer, although a potential benefit was observed in selected subgroups, whereas Nakagawa et al. reported improved outcomes among patients with multiple previous implantation failures [21,22]. A blastocyst-transfer study by Chun et al., however, found no significant improvement in clinical pregnancy or implantation rates with hyaluronan-enriched transfer medium [23]. In frozen embryo transfer cycles, Hambiliki et al. reported an increased implantation rate without a corresponding improvement in delivery rate, while Horiuchi et al. found no significant improvement in clinical pregnancy among low-grade frozen embryo transfers [24,25]. Studies focusing on embryo characteristics have likewise yielded differing findings: Nakagawa et al. reported improved clinical pregnancy rates in morphologically poor euploid blastocyst transfers, whereas a more recent cohort of frozen single euploid blastocyst transfers found no significant overall improvement in reproductive outcomes [26,27]. A recent systematic review and meta-analysis of randomized trials also failed to demonstrate a clear live birth benefit in sensitivity analyses, with the certainty of evidence considered very low [28]. Conversely, a 2026 retrospective cohort of biopsied euploid blastocyst transfers reported higher live birth rates with hyaluronan-enriched transfer medium, although the observational design and potential for residual confounding warrant cautious interpretation [29]. These divergent findings suggest that any potential effect of hyaluronan-enriched transfer media may be context dependent and may not be uniformly observed across different patient populations. Our findings add real-world data from a predominantly frozen, single-blastocyst-transfer cohort comparing a hyaluronan-enriched medium with a lower-hyaluronan medium, in which no statistically significant difference in clinical pregnancy rates was observed.

The retrospective, single-center design of this study introduces several limitations. Although patients were selected according to the study eligibility criteria, the observational nature of the study does not eliminate the possibility of selection bias. Furthermore, the non-randomized allocation of embryo transfer media may have introduced confounding, and residual confounding cannot be excluded despite adjustment for maternal age and transfer type. Importantly, both transfer media contained hyaluronan and differed in other formulation components, making it impossible to isolate the effect of hyaluronan concentration on clinical pregnancy outcomes. Embryo morphology was assessed descriptively but was not included in the multivariable model, and other potentially relevant factors, including endometrial characteristics and transfer-related variables, were not fully accounted for. The relatively small number of patients in the EmbryoGlue group may have limited statistical power to detect clinically meaningful differences. Finally, live birth and long-term obstetric outcomes were not evaluated. Therefore, the findings should be interpreted as associations rather than causal effects, and the absence of a statistically significant difference should not be considered evidence of equivalence between the transfer media.

Despite these limitations, the present study provides real-world evidence from a contemporary cohort restricted to blastocyst-stage embryo transfers, with the majority of cycles performed as frozen embryo transfers. This relatively homogeneous transfer setting may complement previous studies that included more heterogeneous populations with respect to embryo developmental stage and transfer type. Each woman contributed only one transfer cycle, avoiding within-patient clustering. Furthermore, all patients underwent single blastocyst transfer, eliminating potential confounding related to differences in the number of embryos transferred. In addition, the study reflects routine clinical practice rather than a highly selected trial population. Nevertheless, these features should be regarded as methodological strengths rather than evidence of treatment efficacy, and the findings should be interpreted as observational evidence regarding clinical pregnancy outcomes following blastocyst transfer using a hyaluronan-enriched medium versus a lower-hyaluronan medium.

## 5. Conclusions

In this retrospective cohort of single blastocyst transfer cycles, no statistically significant difference in clinical pregnancy rates was observed between the hyaluronan-enriched transfer medium and the lower-hyaluronan transfer medium, including in an exploratory analysis adjusted for maternal age and transfer type. Because both transfer media contain hyaluronan and differ in other formulation components, the findings cannot be attributed specifically to differences in hyaluronan concentration. Given the non-randomized design, residual confounding, unequal group sizes, and limited availability of relevant clinical and embryological covariates, the results should not be interpreted as evidence of equivalence or as demonstrating the absence of a clinically relevant effect. Prospective, adequately powered randomized studies evaluating clinically relevant outcomes, particularly live birth, are warranted.

## Figures and Tables

**Table 1:** Baseline characteristics and clinical pregnancy outcomes.

Variable	EmbryoGlue^®^ (Higher HA; *n* = 72)	SAGE 1-Step^TM^ (Lower HA; *n* = 349)	*p*-Value
Maternal age (years), mean ± SD	33.29 ± 6.76	31.85 ± 5.96	0.068
Fresh embryo transfer, *n* (%)	7 (9.7%)	45 (12.9%)	0.480
Frozen embryo transfer, *n* (%)	65 (90.3%)	304 (87.1%)	—
Single embryo transfer, *n* (%)	72 (100%)	349 (100%)	—
Clinical pregnancy, *n* (%)	21 (29.2%)	127 (36.4%)	0.300

Clinical pregnancy was defined as an intrauterine pregnancy with fetal cardiac activity. All cycles involved single blastocyst transfer. HA, hyaluronan; SD, standard deviation; —, not applicable (no between-group variation or duplicate test).

**Table 2:** Blastocyst expansion stage and morphology.

Variable	EmbryoGlue^®^ (Higher HA; *n* = 72)	SAGE 1-Step^TM^ (Lower HA; *n* = 349)
Expansion stage 3	39 (54.2%)	180 (51.6%)
Expansion stage 4	22 (30.6%)	93 (26.6%)
Expansion stage 5	11 (15.3%)	76 (21.8%)
Morphology AA	27 (37.5%)	119 (34.1%)
Morphology AB	26 (36.1%)	139 (39.8%)
Morphology BB	19 (26.4%)	91 (26.1%)

Values are presented as *n* (%). Blastocyst morphology was assessed using the Gardner grading system. HA, hyaluronan.

**Table 3:** Multivariable logistic regression for clinical pregnancy.

Variable	Adjusted OR	95% CI	*p*-Value
EmbryoGlue^®^ (vs. SAGE 1-Step^TM^)	0.77	0.44–1.34	0.352
Maternal age (per year)	0.95	0.91–0.98	0.002
Frozen (vs. fresh) embryo transfer	1.14	0.59–2.21	0.688

OR, odds ratio; CI, confidence interval. Model adjusted for maternal age and embryo transfer type. SAGE 1-Step^TM^ was the reference transfer medium.

**Table 4:** Clinical pregnancy by maternal age subgroup.

Age Group	EmbryoGlue^®^ (Higher HA), *n*/*N* (%)	SAGE 1-Step^TM^ (Lower HA), *n*/*N* (%)	OR (95% CI)	*p*-Value
≤35 years	14/44 (31.8%)	100/251 (39.8%)	0.71 (0.36–1.40)	0.313
>35 years	7/28 (25.0%)	27/98 (27.6%)	0.88 (0.33–2.30)	0.789

OR, odds ratio; CI, confidence interval; HA, hyaluronan. ORs compare EmbryoGlue^®^ with SAGE 1-Step^TM^ within each age subgroup.

## Data Availability

The datasets generated and/or analyzed during the current study are available from the corresponding author upon reasonable request, subject to institutional approval and applicable patient confidentiality and data protection requirements.
